# Frailty Syndrome and Cardiovascular Diseases in Older People

**DOI:** 10.3390/healthcare13243275

**Published:** 2025-12-13

**Authors:** Gabriela Cristina Chelu, Ovidiu Lucian Băjenaru, Cătălina Raluca Nuță, Lidia Băjenaru, Gabriel Ioan Prada

**Affiliations:** 1 Faculty of Medicine, “Carol Davila” University of Medicine and Pharmacy, 050474 Bucharest, Romania; gabriela-cristina.chelu@umfcd.ro (G.C.C.); catalina.nuta@umfcd.ro (C.R.N.); gabriel.prada@umfcd.ro (G.I.P.); 2 National Institute of Gerontology and Geriatrics “Ana Aslan”, 011241 Bucharest, Romania; 3 Department of Communications, Applications, and Digital System, National Institute for Research and Development in Informatics—ICI Bucharest, 011455 Bucharest, Romania; lidia.bajenaru@ici.ro; 4 Department of Computer Science, Faculty of Automatic Control and Computers, National University of Science and Technology POLITEHNICA Bucharest, 060042 Bucharest, Romania; 5 Academy of Romanian Scientists, 050044 Bucharest, Romania

**Keywords:** frailty syndrome, cardiovascular disease, older people

## Abstract

**Objective:** Cardiovascular diseases have a high prevalence among the elderly, together with frailty syndrome, and both conditions negatively affect quality of life and limit patient autonomy. This study aimed to explore potential relationships between cardiovascular and metabolic parameters, renal function, and frailty domains to identify potential intervention targets. **Methods:** A cross-sectional study was conducted between January 2024 and April 2025 at the National Institute of Gerontology and Geriatrics “Ana Aslan”, including 359 patients aged over 40 years. Demographic, anthropometric, and clinical data were collected through interviews, medical records, and standardized assessments of frailty components (weakness, exhaustion, slow gait, balance impairment, reduced activity, cognitive decline, and weight loss), as well as cardiovascular diseases and comorbidities. **Results:** Most participants were aged 65–79 years. ROC curve identified triglycerides as a good indicator of both alcohol consumption (AUC = 0.631, *p* = 0.042) and smoking status (AUC = 0.676, *p* = 0.004), while HDL cholesterol showed an inverse association with smoking status (AUC = 0.356, *p* = 0.019). Reduced renal function was significantly associated with smoking status, balance, gait impairment, and reduced functional mobility. The Up and Go Test indicated a good discriminatory ability for renal function decline (AUC = 0.656, *p* < 0.001). Muscle strength, MMSE, and Tinetti scores showed inverse associations with renal function. **Conclusions:** Renal impairment appears to be a reliable indicator across multiple frailty domains, acting as an accelerator of frailty progression. Triglycerides reflect lifestyle-related factors, while the Up and Go Test may serve as a practical screening tool for renal dysfunction in frail older adults. These findings suggest the need to adapt traditional cardiovascular risk management to the frail geriatric population.

## 1. Introduction

Frailty is a common and clinically significant syndrome among older adults, characterized by age-related declines in function and physiological reserve across multiple organ systems [1]. This progressive reduction in homeostatic capacity contributes to increased vulnerability to adverse health outcomes. Considered a biological syndrome resulting from multisystem declines in physiological reserves, frailty develops through a multitude of direct, indirect, and interacting risk factors. Due to its clinical development, frailty is easily associated with increased risk of falls, disability, systemic diseases, frequent hospitalizations, and institutionalization. Fried et al. have described frailty as the presence of three out of five vital components: unintentional weight loss, slow walking speed, self-reported exhaustion, low energy expenditure, and weakness [2,3,4].

Cardiovascular diseases affect a large number of people over 60 and are one of the main causes of death in the world. As population aging accelerates and life expectancy rises, age persists as the predominant cardiovascular risk factor for the onset of cardiovascular disease (CVD). From this perspective, we can anticipate a rise in both incidence and prevalence, thereby making CVD a significant social burden in terms of mortality, morbidity, resource utilization, and healthcare costs. Age remains a risk factor for geriatric syndromes such as frailty, sarcopenia, functional disability, and cognitive impairment, which have a major impact on management and outcomes in older adults with CVD [5].

Frailty and CVD are connected, so patients who have been diagnosed with CVD are predisposed to developing frailty, and the presence of frailty accelerates the risk of developing CVD [6].

According to a recent review from 2024 [7], frailty increases vulnerability to disease, particularly CVD, and is characterized by physiological disturbances—including sarcopenia, inflammatory activation, and autonomic dysfunction—that predispose patients to illness and disability. In 2023, López-Otín et al. [8] describe 12 mechanisms of aging, and a few of them were associated with frailty and CVD (e.g., chronic inflammation, metabolic dysregulation, etc.). Chronic inflammation and immune activation are defined by the disproportion between inflammatory and anti-inflammatory pathways, leading to “inflammageing” and frailty. Elevated inflammatory markers, such as interleukin 6 (IL-6), tumor necrosis factor α (TNF- α), and C-reactive protein, are associated with frailty [9,10], but IL-6 has also been associated with CVD [11].

Recent evidence from Central Europe also highlights relevant lifelong determinants of cardiovascular risk. A Slovak study in midlife women showed that early menarche and the absence of breastfeeding were significantly associated with higher odds of obesity and obesity-related hypertension in later life [12]. Another Slovak analysis found that women with cardiovascular disease had markedly higher body mass index and waist–hip ratio values compared with the control group, underscoring the strong link between adiposity and cardiovascular complications in this population [13].

Even so, this area lacks sufficient information and needs further research to determine possible triggers for these pathological changes. Metabolic dysregulation may lead to altered hormone levels, such as low testosterone, insulin resistance, and metabolic syndrome, which are common to both frailty and CVD. A systematic review developed by Jiang et al. showed an important association between metabolic syndrome and frailty in adults [14]. More significantly, the complications within these afflictions independently increase the incidence of frailty and CVD. As proven by Shankya et al., diabetes mellitus is associated with vascular dysfunction, loss of subcutaneous fat, and increased visceral fat, reducing energy expenditure, altering the secretion of sex hormones, and contributing to frailty and CVD [15].

The interaction between the two pathologies has become a subject of increasing interest within the medical community, with the prevalence of frailty syndrome among patients with cardiovascular disease ranging from 25% to 62%. For example, the Pro. V.A. study [16], which followed non-frail participants over the age of 65, showed that various components of frailty, such as low energy expenditure and exhaustion, were highly associated with the onset of CVD, such as heart failure. Though various studies have proven an association between frailty and the incidence of CVD that is not related to the traditional risk factors, both prefrailty and frailty are also associated with multimorbidity, cognitive impairment, and metabolic dysregulation [6,16]. Elderly individuals with frailty are often affected by multimorbidity (≥2 chronic conditions), and multimorbidity itself is associated with a two-fold higher risk of frailty compared with those without multiple chronic diseases [17].

Frailty syndrome may be prevented in patients with cardiovascular disease, and early identification and timely intervention are important to avoid disability, reduced quality of life, and, in some cases, to delay the onset of cardiovascular disease [18].

## 2. Methods

Patients: The study included 359 patients who were admitted to the National Institute of Gerontology and Geriatrics “Ana Aslan”, Bucharest, over the age of 40, between January 2024 and April 2025. All patients had cardiovascular diseases. Exclusion criteria: Patients younger than 40 years, patients without cardiovascular comorbidities, and those who did not agree to participate in the study. Every patient was presented with the study aims and explained the consent form, to make sure that there were no doubts regarding the research protocol, and then the patients were asked to sign the consent form. The protocol was approved by the local ethics committee (protocol code 113, approval date: 23 November 2023, and protocol code 231, approval date: 4 April 2025).

The patients included in the study were divided in 2 groups: a control group of participants aged 40–64 years and a study group of those aged 65 years and older.

Variables: Demographic data (age, sex, smoking, and alcohol consumption), anthropometric data (weight, height) were collected through interviews. Frailty was assessed using the following parameters: slow gait, poor balance, reduced physical activity, cognitive impairment (evaluated with the Mini-Mental State Examination—MMSE [19]), and malnutrition risk (evaluated with the Mini Nutritional Assessment—MNA [20]). We also evaluated and collected the following data: chronic kidney disease (CKD) and other comorbidities (high blood pressure (HBP); type 2 diabetes mellitus (T2DM); presence of dyslipidemia; and atherosclerosis) were collected through interview, personal medical data, and specific tests within the study timeframe. Poor balance and reduced physical activity were defined using the Tinetti Test [21] (a score lower than or equal to 23 points was considered impaired, and a score over 23 points was considered normal). Slow gait was defined using the Up and Go Test [22] (if the time was lower than 10 s, it was considered normal, and if it was over or equal to 10 s, it was considered impaired). Grip strength was assessed using a validated Handgrip Strength Test. Chronic kidney disease [23] was evaluated with the official staging based on estimated glomerular filtration rate (eGFR), calculated using the 2021 CKD-EPI creatinine formula [24] (stage 1 and 2—not affected, and stage 3 and above—affected). Body mass index (BMI) [25] was calculated by dividing weight by square height and was defined as underweight for a BMI lower than 18.5 kg/m^2^, normal weight for a BMI between 18.5 and 24.9 kg/m^2^, overweight for a BMI between 25.0 and 29.9 kg/m^2^, class I obesity for a BMI between 30.0 and 34.9 kg/m^2^, class II obesity (severe obesity) for a BMI between 35.0 and 39.9 kg/m^2^, and class III obesity (morbid obesity) for a BMI over or equal to 40.00 kg/m^2^. HBP was defined in the following ways: if the patient was diagnosed in the past with HBP by a physician and now has controlled blood pressure under medication, or if the patient was not known to have HBP and we diagnosed them based on the values of the blood pressure measurements of at least 140/90 mmHg on the day of admission. T2DM was determined if the patient had a diagnosis of T2DM according to the attending physician, or if the patient was diagnosed on the day of the admission according to the international criteria (at least two recorded fasting plasma glucose levels of at least 126 mg/dL, or by a 2 h plasma glucose level ≥200 mg/dL (11.1 mmol/L) during a standard 75 g oral glucose tolerance test (OGTT) [26], or HbA1c ≥ 6.5%). Dyslipidemia was determined if the patient had a diagnosis of dyslipidemia according to the attending physician, or if the biological evaluation of the patient at admission had recorded a total cholesterol of 230 mg/dL or above, serum low-density lipoproteins of 130 mg/dL or above, or serum triglycerides of 150 mg/dL or more. Atherosclerosis was determined if the patient had a diagnosis of atherosclerosis according to the attending physician or if the patient had a recorded imaging study.

The analysis was performed using IBM SPSS Statistics 20, and the following statistical tests were used: Chi-squared test and correlation coefficients (Kendall’s tau-b and Spearman’s rho), as well as a Receiver Operating Characteristic (ROC) curve analysis.

## 3. Results

The sample included 359 patients. The baseline characteristics of the study population are summarized in Table 1. The mean age distribution showed a predominance of patients in the 65–74 years age group (40.4%), followed by those aged 75–79 years (20.9%). Nearly one-fifth of participants (18.7%) were octogenarians (≥80 years). The study population demonstrated a marked female predominance (82.2% vs. 17.8% male). Most participants resided in urban areas (70.2%) compared to rural settings (29.8%). In terms of lifestyle characteristics, 74.9% of patients did not smoke, whereas 10.9% had previously smoked and 14.2% were current smokers. The cardiovascular risk profile revealed a high prevalence of traditional risk factors. High blood pressure was present in 82.7% of patients, representing the most common comorbidity. Dyslipidemia affected 72.7% of the study population, while type 2 diabetes mellitus was diagnosed in 27.3% of participants, with an additional 1.1% having prediabetes. Atherosclerosis was documented in 20.1% of patients. Chronic kidney disease (stage 3 and above) was present in 20.6% of the study population.

Balance and gait assessment using the Tinetti test revealed a progressive age-related decline in functional performance, as shown in Table 2. Among the youngest participants (≤64 years), 81.9% exhibited normal balance and gait, with only 4.2% demonstrating high fall risk. In the 65–74 years age group, 62.8% maintained normal function, while 13.1% had high fall risk. The functional decline became more pronounced in older participants: among those aged 75–79 years, 49.3% maintained normal balance and gait, with 41.3% showing fall risk. The oldest participants (≥80 years) demonstrated the most severe impairment, with only 32.8% maintaining normal function and 29.9% showing high fall risk, representing a significant increase compared to those under 65 years.

The Up and Go test revealed a progressive deterioration of functional mobility with advancing age (Table 3). Among participants in the ≤64 years age group, the vast majority (88.6%) presented normal mobility, with only 11.4% having impaired mobility. The prevalence of impaired mobility increased significantly in older age groups: 28.0% in those aged 65–74 years, 43.8% in those aged 75–79 years, and 57.1% in participants ≥80 years. Therefore, an approximately five-fold increase in the prevalence of impaired mobility was observed in the oldest participants compared to the youngest group, highlighting the significant impact of aging on functional mobility.

Grip strength assessment revealed a progressive age-related decline in muscle strength across all age groups, as shown in Table 4. In the 65–74 years age group, grip strength decreased to 23.2 kg (right arm) and 20.9 kg (left arm), representing a modest decline from the group ≤64 years. The decline became more pronounced in older patients: in the group aged 75–79 years, mean grip strength was 19.7 kg (right arm) and 18.5 kg (left arm), and in the group aged ≥80 years, mean grip strength was 17.7 kg (right arm) and 16.7 kg (left arm), revealing a severe impairment in the oldest group of patients. Therefore, an approximately 27% decrease in grip strength for the right arm and 25% for the left arm was observed in the oldest group of patients compared to the youngest group, highlighting the significant impact of aging on muscle strength and functional capacity.

**Figure 1:**
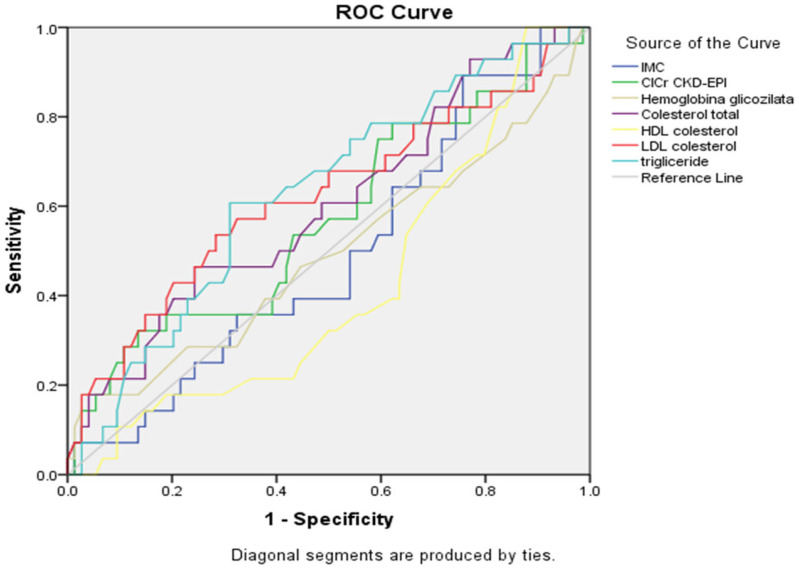
ROC curves for metabolic parameters showing a connection with alcohol consumption status (n = 359).

ROC curve (Figure 1, Table 5) demonstrated that triglycerides had the better association with alcohol consumption (AUC = 0.631, 95% CI: 0.512–0.750, *p* = 0.042), with LDL cholesterol showing a trend toward significance (AUC = 0.621, *p* = 0.060). Total cholesterol demonstrated moderate discriminatory ability, but did not reach statistical significance (AUC = 0.601, *p* = 0.116), and HDL cholesterol showed a trend towards inverse association, but did not reach statistical significance (AUC = 0.420, *p* = 0.216). The ROC curves confirmed these findings, with triglycerides and LDL cholesterol curves showing the greatest deviation from the reference diagonal line. Other metabolic parameters demonstrated poor discriminatory ability, with HbA1c and BMI curves and renal function, with their curves closely following the line of no discrimination.

**Figure 2:**
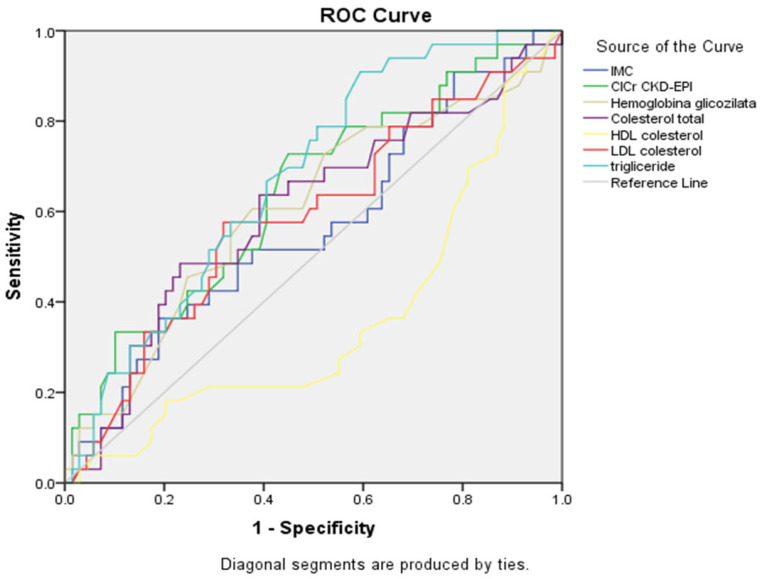
ROC curves for metabolic and renal parameters showing connection with smoking status (n = 359).

The ROC curve (Figure 2, Table 6) showed that triglycerides had the best association with smoking status (AUC = 0.676, 95% CI: 0.571–0.781, *p* = 0.004), followed by renal function assessed by estimated glomerular filtration rate (eGFR) using the CKD-EPI formula (CICr CKD-EPI) (AUC = 0.639, 95% CI: 0.523–0.755, *p* = 0.023).

Notably, HDL cholesterol showed an inverse association (AUC = 0.356, 95% CI: 0.239–0.473, *p* = 0.019), indicating that lower HDL values were associated with smoking status. Among the lipid parameters, total cholesterol showed a trend toward significance (AUC = 0.603, 95% CI: 0.482–0.724, *p* = 0.093), while LDL cholesterol demonstrated moderate, but non-significant, discriminatory ability (AUC = 0.587, *p* = 0.157), but neither reached statistical significance. The ROC curves confirmed these findings, with triglycerides and renal function curves showing the greatest deviation from the reference diagonal line. HbA1c (AUC = 0.606, *p* = 0.085) and BMI (AUC = 0.565, *p* = 0.291) showed minimal discriminatory ability without statistical significance.

**Figure 3:**
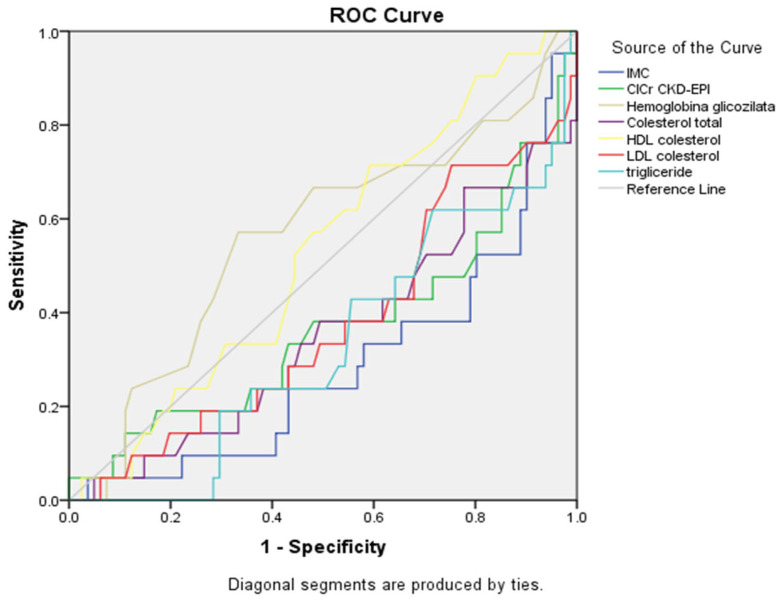
ROC curves for metabolic and renal parameters showing connection with cognitive impairment (MMSE) (n = 359).

ROC curve (Figure 3, Table 7) for cognitive impairment (MMSE) prediction revealed that several metabolic parameters demonstrated significant inverse associations with cognitive function. BMI showed the strongest inverse association (AUC = 0.287, 95% CI: 0.160–0.414, *p* = 0.003), indicating that higher BMI values were significantly associated with cognitive impairment. Triglycerides also demonstrated a significant inverse relationship (AUC = 0.330, 95% CI: 0.203–0.456, *p* = 0.016), followed by total cholesterol (AUC = 0.352, 95% CI: 0.214–0.491, *p* = 0.038). Renal function showed a trend toward inverse association (AUC = 0.366, 95% CI: 0.218–0.515, *p* = 0.060), suggesting that reduced renal function may be linked to cognitive decline, while LDL cholesterol demonstrated a similar trend (AUC = 0.375, *p* = 0.079). The ROC curves confirmed these findings, with BMI, triglycerides, and total cholesterol curves positioned below the reference diagonal line, indicating inverse associations. In contrast, HbA1c (AUC = 0.564, *p* = 0.367) and HDL cholesterol (AUC = 0.530, *p* = 0.676) showed minimal discriminatory ability, with their curves closely following the reference line, without reaching statistical significance.

**Figure 4:**
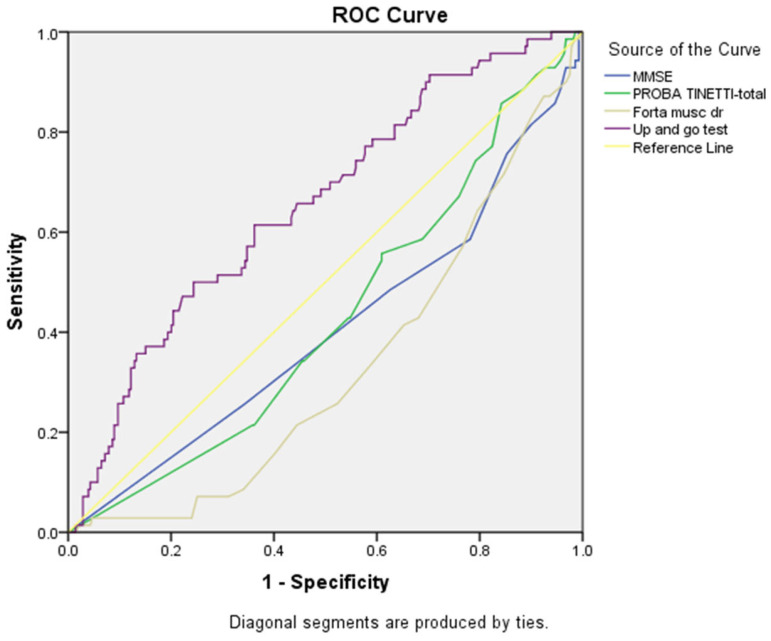
ROC curves for cognitive and frailty assessment tools showing connection with chronic kidney disease (CKD) (n = 359).

ROC curve (Figure 4, Table 8) for chronic kidney disease (CKD) prediction demonstrated that the Up and Go Test had the strongest discriminatory ability (AUC = 0.656, 95% CI: 0.585–0.726, *p* < 0.001), showing a good ability to identify CKD. Muscle strength also demonstrated significant discriminatory ability with an inverse association (AUC = 0.329, 95% CI: 0.263–0.394, *p* < 0.001), indicating that reduced muscle strength was strongly associated with CKD presence. MMSE showed a significant inverse relationship (AUC = 0.405, 95% CI: 0.328–0.483, *p* = 0.014) while the Tinetti test demonstrated a trend toward inverse association (AUC = 0.428, 95% CI: 0.356–0.500, *p* = 0.063). The ROC curves confirmed these findings, with the Up and Go Test curve showing the greatest deviation from the reference diagonal line, while muscle strength, MMSE, and Tinetti curves were positioned below the reference line. These inverse relationships indicate that poorer performance on these functional assessments was associated with a higher risk of CKD, suggesting an association relationship between renal function, frailty syndrome, and cognitive impairment.

**Figure 5:**
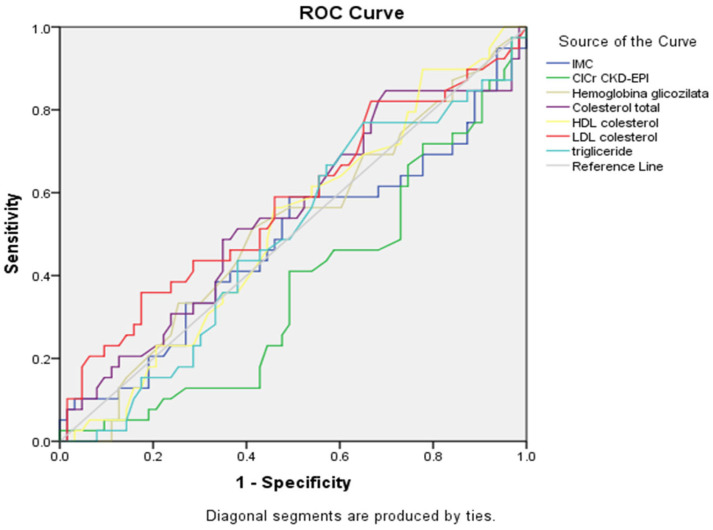
ROC curves for metabolic and renal parameters showing connection with balance and gait impairment (Tinetti test) (n = 359).

ROC curve (Figure 5, Table 9) for balance and gait impairment (Tinetti test) prediction showed that renal function demonstrated a significant inverse association (AUC = 0.368, 95% CI: 0.258–0.478, *p* = 0.026), indicating that reduced renal function was associated with increased risk of balance and gait dysfunction. Among lipid parameters, LDL cholesterol showed the highest discriminatory ability (AUC = 0.575, 95% CI: 0.458–0.693, *p* = 0.203), followed by total cholesterol (AUC = 0.546, 95% CI: 0.428–0.663, *p* = 0.439), though neither reached statistical significance. The ROC curves confirmed these findings, with eGFR positioned below the reference diagonal line, demonstrating the inverse relationship. The remaining metabolic parameters showed minimal discriminatory ability, with HbA1c (AUC = 0.514, *p* = 0.810), HDL cholesterol (AUC = 0.515, *p* = 0.794), triglyceride (AUC = 0.490, *p* = 0.866), and BMI (AUC = 0.479, *p* = 0.720) curves closely following the line of no discrimination.

**Figure 6:**
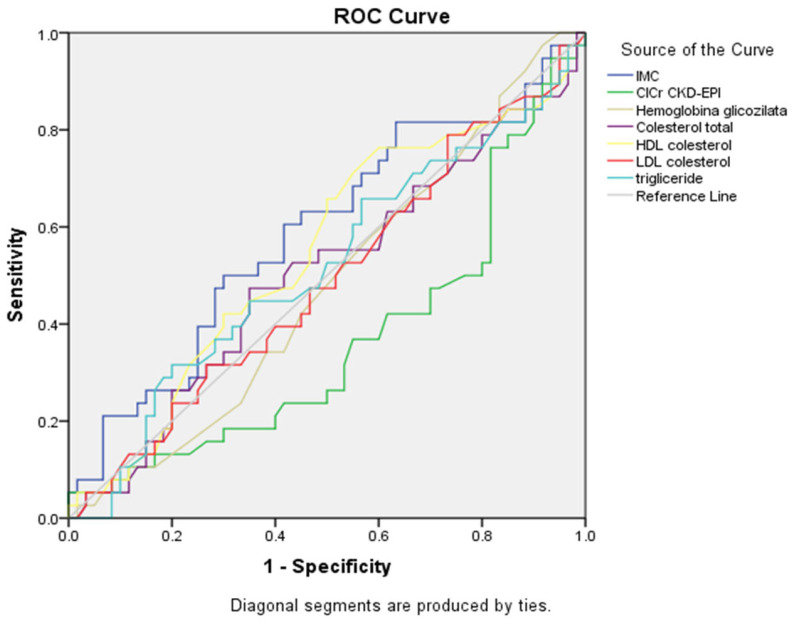
ROC curves for metabolic and renal parameters showing connection with functional mobility impairment (Up and Go Test) (n = 359).

ROC curve (Figure 6, Table 10) for functional mobility impairment (Up and Go Test) prediction showed that renal function demonstrated a significant inverse association with functional mobility (AUC = 0.363, 95% CI: 0.248–0.479, *p* = 0.023), indicating that reduced renal function was associated with increased risk of mobility impairment. Among other parameters, BMI had the highest discriminatory ability (AUC = 0.588, 95% CI: 0.470–0.706, *p* = 0.143), followed by HDL cholesterol (AUC = 0.548, 95% CI: 0.429–0.666, *p* = 0.429), though neither reached statistical significance.

The ROC curves confirmed these findings, with eGFR positioned below the reference diagonal line, demonstrating the inverse relationship, while BMI showed a better positive deviation from the reference line. The remaining metabolic parameters demonstrated minimal discriminatory ability, with triglycerides (AUC = 0.516, *p* = 0.796), total cholesterol (AUC = 0.503, *p* = 0.962), LDL cholesterol (AUC = 0.491, *p* = 0.884), and HbA1c (AUC = 0.479, *p* = 0.721) curves closely following the line of no discrimination.

## 4. Discussion

This study, which included a total of 359 hospitalized elderly patients (predominantly aged 65–79 years), examined the interrelationships between metabolic parameters, renal function, lifestyle factors, and frailty syndrome, assessed using multiple validated frailty-related scales and functional assessments.

The ROC curves identified triglycerides as a good indicator of both alcohol consumption (AUC = 0.631, *p* = 0.042) and smoking status (AUC = 0.676, *p* = 0.004), demonstrating moderate discriminatory ability. These results align with the observations of Wakabayashi et al. [27] who reported similar triglyceride elevations in elderly alcohol consumers.

The inverse association between HDL cholesterol and smoking status (AUC = 0.356, *p* = 0.019) is consistent with previous studies [18], which reported persistent HDL suppression in elderly smokers.

Our results revealed a significant association between affected renal function assessed by estimated glomerular filtration rate (eGFR) using the CKD-EPI formula (CICr CKD-EPI) and smoking status (AUC = 0.639, *p* = 0.023). Through the ROC curve, the findings from this study extend smoking as an independent risk factor for CKD progression to hospitalized elderly patients, as proven by researchers [28].

Our study showed inverse associations between metabolic parameters (BMI (AUC = 0.287, *p* = 0.003), triglycerides (AUC = 0.330, *p* = 0.016), and total cholesterol (AUC = 0.352, *p* = 0.038)) and cognitive function (MMSE). These findings present an unexpected pattern and require careful interpretation. Recent studies have documented this unexpected pattern, such as the PreDIVA trial conducted by Schroevers et al. [28], which showed that dyslipidemia (especially high LDL/HDL ratio) was associated with lower dementia risk in adults aged 70–78 years, with a pronounced effect on individuals with low BMI and higher education. Chen et al. [29], studying oldest-old and centenarian adults, found that the participants enrolled in the study with cognitive impairment had significantly lower cholesterol levels, suggesting that higher cholesterol levels may be protective in very old age. Isong et al. showed a high prevalence of metabolic syndrome and cognitive impairment among older adults from Calabar Metropolis, with an important association between lower HDL levels and poor cognitive status. This study emphasizes the importance of managing specific metabolic risk factors, particularly HDL cholesterol, to preserve cognitive health in the aging population [30]. Therefore, these findings suggest metabolic dysfunction and cognitive decline may represent parallel manifestations of systemic frailty, rather than direct causation.

This trend toward inverse association between renal function and cognitive decline (AUC = 0.366, *p* = 0.060) is consistent with the kidney–brain axis concept, explained by Bugnicourt et al. [31], though this is weaker than the findings in the cross-sectional REGARDS study by Kurella et al. [32], which found an 11% increased prevalence of cognitive impairment for each 10 mL/min/1.73 m^2^ decrease in eGFR in a large cohort of US adults with CKD. Hafez et al. [33] suggested that re-evaluating drug prescription for CKD patients may represent an important decision in preventing or improving CKD-related cognitive impairment, due to the different effects that these drugs have on cognitive impairment. Drew et al. [34] recommend that CKD patients should use drugs that may delay the progression of cognitive impairment, in addition to treating kidney diseases, such as ACEIs/ARBs, SGLT2i, antidiabetic agents, and antihypertensive agents. Hafez et al. recommend reducing or avoiding the use of drugs that may aid the progression of cognitive impairment, such as antibiotics, anticholinergics, and opioids [33].

Due to the connection between the brain and kidneys, patients with cognitive impairment might require monitoring of their renal function [35].

Renal function assessed by estimated glomerular filtration rate (eGFR) using the CKD-EPI formula (CICr CKD-EPI) consistently predicted multiple frailty domains: balance and gait impairment (AUC = 0.368, *p* = 0.026) and functional mobility (AUC = 0.363, *p* = 0.023). The findings in our study support Musso et al.’s work [36] characterizing renal function as a frailty accelerator. The results of our study concur with Roshanravan et al.’s [37] theory that reduced kidney function independently associates with decreased physical performance. Even so, moderate AUC values of the ROC curve (Figure 5) indicate that renal function explains only a partial difference, consistent with the multifactorial nature of frailty syndrome [18].

The minimal discriminatory ability of the metabolic parameters for physical frailty (AUC = 0.5) is at odds with Barzilay et al.’s Cardiovascular Health Study findings [38], possibly indicating the differences in hospital versus community settings or assessment methods.

The most relevant finding in our study shows associated relationships: the Up and Go Test showed a good discriminatory ability for renal function decline (AUC = 0.656, *p* < 0.001), while muscle strength (AUC = 0.329, *p* < 0.001), MMSE (AUC = 0.656, *p* < 0.014), and Tinetti tests showed associations with inverse renal function decline. This finding concurs with the “vicious cycle” theory described by Chang et al. [39], where frailty and cognitive impairment interact to increase the risk of adverse outcomes (disability, falls), which, in our study, revealed as an associated relationship between renal function decline and physical performance measurements (Up and Go Test, Tinetti Test, and muscle strength), as well as cognitive function (MMSE).

The novelty of our study lies in the Up and Go Test’s effectiveness in showing a connection with renal function decline, with significant clinical implications. While previous studies examined frailty as a consequence of renal function decline, as shown by Nixon et al. [40], fewer studies evaluated functional assessments as renal function decline screening tools. Our findings suggest that this inexpensive and widely available tool could identify hospitalized elderly patients in need of a nephrological evaluation.

The inverse associations between functional measurements and renal function decline align with the nature of frailty syndrome, suggesting that renal function decline accelerates multiple frailty domains.

Conventional cardiovascular risk factors (lipid profile, BMI) showed complex relationships with frailty outcomes, aligning with the “risk factor paradox” described by Afilalo et al. [41], where conventional risk factors may lose predictive value in the frail elderly population. Two recent systematic reviews and meta-analyses provide strong evidence for supporting the age-dependent relationship. Dao et al.’s findings [42] confirmed significant associations between metabolic syndrome and frailty in older adults, while marking substantial heterogeneity in effect sizes across age groups. In their European meta-analysis, Jiang et al. [14] reported similar results, documenting that metabolic syndrome components show varying strength of association with frailty, depending on age, with cardiovascular risk paradigms becoming less applicable in the oldest-old. These results suggest that the population in our study of ages between 65 and 79 years represents a transitional stage in which traditional cardiovascular paradigms become less relevant. A recent study performed in Japan emphasized the importance of assessing comprehensive frailty-related indicators (physical function, nutritional status, and cognitive function) next to traditional risk factors, in order to more accurately evaluate the risk of CVD in older adults [43].

Liu et al. showed in a recent study that sustained frailty remission was highly associated with a lower risk of CVD, in comparison with people who were persistently pre-frail or frail. This study emphasizes the dynamic and likely reversible nature of frailty, indicating that remission of frailty may offer long-term cardiovascular benefits [44].

Johnson et al. suggest in their recent study that a standardized frailty assessment in cardiovascular care is highly needed. They also recommend integrating frailty into CVD guidelines and, therefore, embracing multidisciplinary care models, in order to improve risk stratification and individualize treatment [45].

Although metabolic parameters alone showed limited utility in showing a connection with physical frailty, they remain valuable within a comprehensive assessment of the patient, as their associations with lifestyle factors and cognitive function reflect overall health status.

### 4.1. Unresolved Problems and Future Research

Further elucidation of the molecular mechanisms underlying the kidney–brain–muscle axis is warranted. It remains unclear which of the major factors—uremic toxins, systemic inflammation, or vascular dysfunction—plays the predominant role in mediating this interaction. Future studies incorporating inflammatory markers, such as IL-6 and CRP, could help clarify these pathways and potentially identify novel therapeutic targets.

The Up and Go Test’s clinical utility for renal dysfunction screening needs clinical validation. Side effects of medication, such as ACE inhibitors, statins, or antidiabetic agents, need further investigation to determine whether targeting metabolic or renal parameters can prevent the progression of frailty.

Also, moderate AUC values suggest that additional unmeasured factors, such as genetics, lifetime exposure, and social determinants, might play a significant role, requiring a much more comprehensive approach.

The results should be interpreted in the context of the underlying cardiovascular impairment present in the study cohort. To enhance the generalizability of these findings, future research should consider including patients without cardiovascular pathology, allowing for a clearer assessment of whether the observed associations persist in populations without such comorbidities.

### 4.2. Study Limitations

The study cohort displayed a marked sex imbalance, as female patients are generally more likely to seek medical care than male patients. This inherent characteristic of the evaluated population resulted in an overrepresentation of women in the sample. Consequently, sex-stratified analyses did not reach statistical significance, and this limitation should be considered when interpreting the results.

Despite providing insightful viewpoints, we must interpret the findings within the framework of a cross-sectional study, and therefore, a longitudinal investigation must be carried out in the future to more accurately determine causality.

## 5. Conclusions

This study demonstrated complex associative relationships between metabolic parameters, renal function, lifestyle factors, and frailty syndrome assessed using multiple validated frailty-related scales and functional assessments.

Triglycerides appear to be a good indicator of both alcohol consumption and smoking status, supporting their utility as metabolic markers of lifestyle factors in the elderly population. Also, their moderate discriminatory ability suggests that they should be interpreted within a comprehensive clinical assessment, rather than being used as a standalone screening tool.

Metabolic parameters showed unexpected inverse associations with cognitive function, and, in contrast, higher BMI, triglycerides, and total cholesterol correlated with poorer cognitive performance.

Renal function proved to be a reliable indicator across multiple frailty domains, showing a significant association with smoking status, balance and gait impairment, and functional mobility. These findings position chronic kidney disease as a central contributor to frailty syndrome, supporting the concept of chronic kidney disease as an accelerator of aging and functional decline.

The Up and Go Test showed a good discriminatory ability for identifying chronic kidney disease, becoming a potentially valuable screening tool in hospital settings. Combined with the inverse associations between muscle strength, cognitive function, gait, and balance, and in the presence of chronic kidney disease, they suggest that the routine frailty assessment could identify elderly patients in need of a nephrology evaluation.

Functional tests like the Up and Go Test could serve as a potential chronic kidney disease screening tool in routine elderly care. Lifestyle interventions targeting smoking and alcohol consumption remain relevant, even in the elderly population, though traditional cardiovascular risk management strategies may require adjustments in the frail elderly population.

## Figures and Tables

**Table 1:** General characteristics of the sample (n = 359).

Characteristic	Category	Total (N = 359) n (%)	Mean ± SD
Age	≤64 years	72 (20.1)	71.52 ± 8.944
	65–74 years	145 (40.4)	
	75–79 years	75 (20.9)	
	≥80 years	67 (18.7)	
Sex	Female	295 (82.2)	-
Residence	Urban	252 (70.2)	-
Smoking status	No	269 (74.9)	-
	Yes	51 (14.2)	-
	Former	39 (10.9)	-
Alcohol consumption	No	247 (68.8)	-
	Yes	25 (7.0)	-
	Occasional	87 (24.2)	-
BMI	Obesity	123 (34.3)	29.86 ± 5.472
MNA	Normal	289 (80.6)	-
Dyslipidemia	Yes	261 (72.7)	-
Atherosclerosis	Yes	72 (20.1)	-
T2DM	Yes	98 (27.3)	-
	Prediabetes	4 (1.1)	-
HBP	No	62 (17.3)	-
Chronic kidney disease	From stage 3 and above	74 (20.6)	-

**Table 2:** Distribution of Tinetti scores by age groups in assessed patients (n = 359).

Tinetti Score	≤64 Years n (%)	65–74 Years n (%)	75–79 Years n (%)	≥80 Years n (%)	*p*-Value
Normal balance and gait	59 (81.9%)	91 (62.8%)	37 (49.3%)	22 (32.8%)	*p* < 0.001
Fall risk present	10 (13.9%)	35 (24.1%)	31 (41.3%)	25 (37.3%)	*p* < 0.001
High fall risk	3 (4.2%)	19 (13.1%)	7 (9.3%)	20 (29.9%)	*p* < 0.001

**Table 3:** Distribution of Up and Go test results by age groups in assessed patients (n = 359).

Up and Go Test	≤64 Years n (%)	65–74 Years n (%)	75–79 Years n (%)	≥80 Years n (%)	*p*-Value
Impaired mobility	8 (11.4%)	40 (28.0%)	32 (43.8%)	36 (57.1%)	*p* < 0.001
Normal mobility	62 (88.6%)	103 (72.0%)	41 (56.2%)	27 (42.9%)	*p* < 0.001

**Table 4:** Distribution of grip strength (mean) by age groups in assessed patients (n = 359).

Grip Strength (Mean ± SD)	≤64 Years	65–74 Years	75–79 Years	≥80 Years	*p*-Value
Right arm	24.4 (±8.9)	23.2 (±8.9)	19.7 (±7.8)	17.7 (±7.6)	*p* < 0.001
Left arm	22.4 (±9.8)	20.9 (±8.2)	18.5 (±8.4)	16.7 (±8.6)	*p* < 0.001

**Table 5:** Summary table for ROC curves (Figure 1).

Variable(s)	AUC	Std. Error	*p*-Value	Asymptotic 95% Confidence Interval	
				Lower Bound	Upper Bound
IMC	0.492	0.063	0.896	0.369	0.614
ClCr CKD-EPI	0.572	0.066	0.262	0.443	0.701
Hemoglobina glicozilata	0.492	0.069	0.902	0.356	0.628
Cholesterol total	0.601	0.064	0.116	0.476	0.726
HDL cholesterol	0.420	0.062	0.216	0.300	0.541
LDL cholesterol	0.621	0.067	0.060	0.490	0.751
Triglyceride	0.631	0.061	0.042	0.512	0.750

**Table 6:** Summary table for ROC curves (Figure 2).

Variable(s)	AUC	Std. Error	*p*-Value	Asymptotic 95% Confidence Interval	
				Lower Bound	Upper Bound
IMC	0.565	0.062	0.291	0.444	0.686
ClCr CKD-EPI	0.639	0.059	0.023	0.523	0.755
Hemoglobina glicozilata	0.606	0.062	0.085	0.484	0.727
Cholesterol total	0.603	0.062	0.093	0.482	0.724
HDL cholesterol	0.356	0.060	0.019	0.239	0.473
LDL cholesterol	0.587	0.062	0.157	0.466	0.708
Triglyceride	0.676	0.054	0.004	0.571	0.781

**Table 7:** Summary table for ROC curves (Figure 3).

Variable(s)	AUC	Std. Error	*p*-Value	Asymptotic 95% Confidence Interval	
				Lower Bound	Upper Bound
IMC	0.287	0.065	0.003	0.160	0.414
ClCr CKD-EPI	0.366	0.076	0.060	0.218	0.515
Hemoglobina glicozilata	0.564	0.074	0.367	0.419	0.709
Cholesterol total	0.352	0.071	0.038	0.214	0.491
HDL cholesterol	0.530	0.066	0.676	0.400	0.660
LDL cholesterol	0.375	0.070	0.079	0.237	0.513
Triglyceride	0.330	0.064	0.016	0.203	0.456

**Table 8:** Summary table for ROC curves (Figure 4).

Variable(s)	AUC	Std. Error	*p*-Value	Asymptotic 95% Confidence Interval	
				Lower Bound	Upper Bound
MMSE	0.405	0.040	0.014	0.328	0.483
PROBA TINETTI-total	0.428	0.037	0.063	0.356	0.500
Forta musc dr	0.329	0.033	0.000	0.263	0.394
Up and go test	0.656	0.036	0.000	0.585	0.726

**Table 9:** Summary table for ROC curves (Figure 5).

Variable(s)	AUC	Std. Error	*p*-Value	Asymptotic 95% Confidence Interval	
				Lower Bound	Upper Bound
IMC	0.479	0.061	0.720	0.359	0.599
ClCr CKD-EPI	0.368	0.056	0.026	0.258	0.478
Hemoglobina glicozilata	0.514	0.059	0.810	0.398	0.630
Cholesterol total	0.546	0.060	0.439	0.428	0.663
HDL cholesterol	0.515	0.058	0.794	0.402	0.629
LDL cholesterol	0.575	0.060	0.203	0.458	0.693
Triglyceride	0.490	0.059	0.866	0.375	0.605

**Table 10:** Summary table for ROC curves (Figure 6).

Variable(s)	AUC	Std. Error	*p*-Value	Asymptotic 95% Confidence Interval	
				Lower Bound	Upper Bound
IMC	0.588	0.060	0.143	0.470	0.706
ClCr CKD-EPI	0.363	0.059	0.023	0.248	0.479
Hemoglobina glicozilata	0.479	0.059	0.721	0.363	0.594
Cholesterol total	0.503	0.061	0.962	0.383	0.623
HDL cholesterol	0.548	0.061	0.429	0.429	0.666
LDL cholesterol	0.491	0.060	0.884	0.373	0.609
Triglyceride	0.516	0.061	0.796	0.396	0.635

## Data Availability

The datasets presented in this article are not readily available because the authors do not own the database used in the presented statistics. They requested access to the database from the owner, the institution that conducted the study. The owner decided to keep the database private.
